# A Pyramid Semi-Autoregressive Transformer with Rich Semantics for Sign Language Production

**DOI:** 10.3390/s22249606

**Published:** 2022-12-08

**Authors:** Zhenchao Cui, Ziang Chen, Zhaoxin Li, Zhaoqi Wang

**Affiliations:** 1 Hebei Machine Vision Engineering Research Center, School of Cyber Security and Computer, Hebei University, Baoding 071002, China; 2 Institute of Computing Technology, Chinese Academy of Sciences, Beijing 100190, China

**Keywords:** human pose generation, sign language production, semi-autoregressive transformer, deep learning

## Abstract

As a typical sequence to sequence task, sign language production (SLP) aims to automatically translate spoken language sentences into the corresponding sign language sequences. The existing SLP methods can be classified into two categories: autoregressive and non-autoregressive SLP. The autoregressive methods suffer from high latency and error accumulation caused by the long-term dependence between current output and the previous poses. And non-autoregressive methods suffer from repetition and omission during the parallel decoding process. To remedy these issues in SLP, we propose a novel method named Pyramid Semi-Autoregressive Transformer with Rich Semantics (PSAT-RS) in this paper. In PSAT-RS, we first introduce a pyramid Semi-Autoregressive mechanism with dividing target sequence into groups in a coarse-to-fine manner, which globally keeps the autoregressive property while locally generating target frames. Meanwhile, the relaxed masked attention mechanism is adopted to make the decoder not only capture the pose sequences in the previous groups, but also pay attention to the current group. Finally, considering the importance of spatial-temporal information, we also design a Rich Semantics embedding (RS) module to encode the sequential information both on time dimension and spatial displacement into the same high-dimensional space. This significantly improves the coordination of joints motion, making the generated sign language videos more natural. Results of our experiments conducted on RWTH-PHOENIX-Weather-2014T and CSL datasets show that the proposed PSAT-RS is competitive to the state-of-the-art autoregressive and non-autoregressive SLP models, achieving a better trade-off between speed and accuracy.

## 1. Introduction

Aiming to improve the communication quality for the deaf community, sign language production (SLP) becomes a key research area in natural language processing. The purpose of SLP is to translate spoken language sentences into the corresponding continuous sign language videos which use graphic humanoid video to express gesture movement. SLP can be divided into two parts: Text to Pose (text2pose), also known as sign poses sequence generation, and Pose to Video (pose2video) which means generating humanoid sign language video from the poses sequence. Since of the importance in SLP, in this paper, we focus on the task of text2pose and propose a novel model to realize an efficient sign poses sequence production.

With the development of deep learning approaches, previous studies [1,2,3,4] have focused on the sequence-to-sequence (Seq2Seq) models based on recurrent neural networks (RNN) [1] or transformers [2,3,4]. The performance of these methods are evaluated by two crucial aspects: (1) the accuracy of predicted sign poses sequence; (2) the network latency during inferring the target sequences.

For the existing RNN-based model [1], the output of the previous node is required to undertake the computation over the present sign pose. Due to the influence of a node is solely in a local range, the information tends to get lost on too long sequences. Another major drawback of RNN-based architecture is the complexity of training. The recurrent property means that it takes a lot of time and computational expense. In contrast, models based on multi-head self attention such as Progressive Transformer (PT) [2], PT&MDN [3], GEN-OBT [5] and MoMP [4] can aggregate information from the global sequences and allow for significantly more parallelization to generate each sign pose. Such approaches can address the vanishing gradients problems when processing long sequences, however, they still suffer from the issues of error accumulation and heavy latency during decoding target frames. The main reason for this is that the decoder with Autoregressive (AT) mechanism generates each frame by conditioning on the previously produced frames.

Unlike the AT models of sequentially generating frames, the models based on Non-Autoregressive (NAT) simultaneously generate entire target sequences. Instead of using the produced frames, the inputs of NAT decoders are entire signals which are independent with the target sequence. Actually, for sign language production, the close neighbors of the current frame have insignificant impact on the final results. Several existing SLP methods have been proposed based on NAT [6,7]. However, such NAT methods have suffered from duplicate frames and missing frames in the generated sequences, which is caused by excessively ignoring the dependencies hidden in the target sequence.

Considering the trade-off between speed and accuracy, Semi-Autoregressive (SAT) decoding scheme [8] was first proposed for neural machine translation. SAT decodes the target sequence by dividing it into multiple groups with a fixed number and then using group-level chain rule instead of word-level chain rule. Inside each group, sign poses are generated in NAT scheme. In the meanwhile, AT property is still maintained between all groups. This mechanism enables SAT to retain the advantages of both AT and NAT. In this work, we find that building the different decoding layers of the SAT via a coarse-to-fine pyramid structure can yield faster and more accurate results.

**Figure 1:**
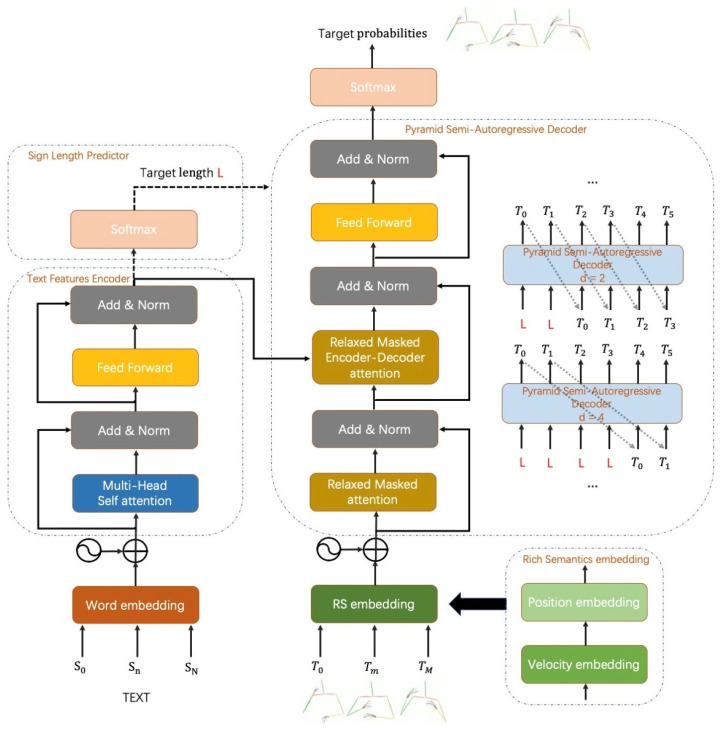
The overall architecture of PSAT-RS, which composes of a text feature encoder with sign length predictor and a pyramid semi-autoregressive decoder with RS embedding.

Based on SAT, in this paper we propose a sigh language production method named Pyramid Semi-Autoregressive Transformer with Rich Semantics (PSAT-RS). As shown in Figure 1, PSAT-RS is composed of three parts: the pyramid SAT (PSAT), sign length predictor and rich semantics embedding layer (RS). One of the core of PSAT is the relaxed masked attention mechanism, which keeps the decoding method of SAT and allows the model to be executed efficiently in a parallel manner. The other core is coarse-to-fine group sizes in the optimal pyramid structure at different layers. Next, the sign length predictor predicts the length of the target sequence in order to serve as input for the first group of PSAT and calculate the number of groups. Lastly, in the existing SLP models such as PT [2] and NAT-GS [6], the feature extraction of sign sequence is only carried out on the time dimension. In contrast, our RS explores the information both on time dimension and spatial displacement by encoding them into the same high-dimensional space.

The main contributions of our work can be summarized as follows:

- We introduce a novel Semi-Autoregressive SLP model, PSAT-RS, which has the autoregressive property in global and generates sign pose concurrently in local.
- To produce realistic and accurate sign pose sequences, a RS embedding module is presented to exploit the spatial displacements and temporal correlations at frame level.
- Experiments demonstrate the superior performance of our method to the competing methods on RWTH-PHOENIX-Weather-2014T dataset and Chinese sign language dataset.

## 2. Related Work

In this section, we discuss existing human action recognition, sign language translation, sign language production methods and transformer based models, among which PT [2], SGN [9] and SAT [8] inspire us to propose the PSAT-RS for SLP.

### 2.1. Human Action Recognition

Skeleton-based human action recognition has been widely attracting a lot of attention in recent years. Recurrent neural networks can find the relationships contained in joint sequences, so researchers [10,11,12] began to use it to cope with temporal dynamics. However, it is very important to explore a more robust long-term spatio-temporal relationship. A recent study by Zhang et al. [9] introduced high-level semantics such as joint types and frame indices into Graph Convolutional Networks (GCN) and Convolutional Neural Networks (CNN). To enhance the capability of feature representation, all frames are associated in self-attention based method by Cho et al. [13]. In their work, temporal information is also exploited by extracting joint-level motion between adjacent frames, which aims at complementing spatio-temporal features.

### 2.2. Sign Language Translation

Specific to continuous sign language translation (SLT), it is a similar task with human action recognition. Necati et al. [14] first introduced the SLT problem which aims to generate spoken language translations from sign language videos. Zhao et al. [15] learnt the spoken language grammar from a large corpus of text sentences and then selected the translation result to the input sign video. Cui et al. [10] proposed a CNN-LSTM network which uses 3D CNN to extract spatial features from each frame of sign language video, and LSTM to generate a possible sentence by analyzing the feature sequence. Due to the weakness in dealing with long-range temporal dependencies, Pu et al. [16] used dilated convolutions with Connectionist Temporal Classification (CTC) loss to approximate the association between different sign words. Considering the original structure, semantics and other characteristics contained in the skeletal data, Tang et al. [17] proposed the Skeleton-GCN model to learn the spatial characteristics of skeleton joints, and built the graph topology representation according to body connectivity. Recent work by Necati et al. [18], has used a transformer-based SLT model and achieved state-of-the-art results. In this paper, we employ this transformer based SLT model [18] as back translation evaluation method to calculate the accuracy of our produced sign language videos.

### 2.3. Sign Language Production

In recent years, most studies in SLP emphasized the use of deep learning methods, such as Recurrent Neural Network (RNN) [1], Generative Adversarial Network (GAN) [19,20,21], and Transformer [2,5]. The first deep learning approach of SLP was proposed by Stoll et al. [22] in 2020, which incorporates RNN structure with GAN. Based on RNN, Xiao et al. [1] proposed a two-level probability skeleton generation model composed of variational autoencoder and gaussian mixed model. The first-level is used to generate random sign poses, and the second-level makes the results close to the real style in nature. However, such RNN-based models are limited to the local inference window and flawed by incomplete feature extraction.

To solve this issue, Saunders et al. [2,3] proposed an autoregressive transformer with a counter to track the decoding progress. Tang et al. [5] focused on the accuracy of pose generation. They designed a further CTC-based back translation model to guarantee the semantic consistency. However, such approaches produce each frame by conditioning on previously generated frames, which lead to error accumulation and heavy latency during inference. Hwang et al. [6] used a non-autoregressive transformer to output all frames in parallel at a more efficient decoding speed. Since it excessively abandons the dependence between sign poses, the issues about repetition and omission are inevitable. In contrast, our method makes a better trade-off between AT and NAT which improves both accuracy and speed of inference.

### 2.4. AT vs. NAT vs. SAT

Most of the neural machine translation models [23,24,25] are autoregressive (AT), generating each token by conditioning on the previous tokens. In order to obtain global contextual information, Vaswani et al. [25] proposed the AT-based transformer, which adopts the self-attention mechanism without convolution. AT-based transformer can obtain the entire target translation during the training phase, each input token of the decoder is the previous ground truth token. However, it still creates a bottleneck at inference stage, since without ground truth, each word generated by the AT decoder is dependent on all previous words. This could lead to error accumulation and heavy latency. Therefore, non-autoregressive (NAT) transformers [26,27] were proposed, which remove the AT connection directly and generate all target words in parallel. While the inference speed is improved, they often suffer from word repetition or omission problem due to removing words dependence excessively.

To make a better balance between speed and quality, Wang et al. [8] proposed a semi-autoregressive (SAT) transformer for retrieving sequential information. The SAT-based transformers [8,28] maintain AT property in global but NAT in local, so that multiple consecutive words can be generated in parallel at each time step. Inspired by these works, we propose a Pyramid Semi-Autoregressive Transformer with coarse-to-fine levels to generate the target sign poses sequence at group-level.

## 3. Methodology

In this section, we will introduce technical details of our proposed PSAT-RS for SLP. The overview of our proposed method is shown in Figure 1. Source spoken language sentence are expressed as $S=(s_{1},\ldots ,s_{N})$ with *N* words and target sign poses sequence $T=(t_{1},\ldots ,t_{M})$ with *M* frames. Our goal is to fit our model of maximizing the computation of conditional probability P(T|S) for sign language production. This section has been divided into three parts. Firstly, we analyze the Rich Semantics (RS) embedding module for feature extraction of sign poses sequence. Then we introduce the relaxed masked attention mechanism to capture correlations between frames within the current group and frames produced in previous groups. Finally, we introduce the details of our network architecture and show how to use the PSAT mechanism with a sign length predictor to output target sequences.

### 3.1. Rich Semantics Embedding Module

Due to the small memory occupation of skeleton data, we use skeleton as the basic unit of our sign language production. In the embedding layer, we respectively map the high-dimensional source spoken language sentence *S* and target sign poses sequence *T* to the low-dimensional feature. Training costs are reduced by automatically learning the mapping from the input raw data to the distributed representation space.

We first embed the source *S* into feature vector $\tilde{S}$ with word embedding module which consists of two linear transformations with a ReLU activation in between as Equation (1)

(1) $$\begin{array}{c} \tilde{S_{n}}=\;relu\left(w_{2}^{s}\left(relu\left(w_{1}^{s}S_{n}+b_{1}^{s}\right)\right)+b_{2}^{s}\right)+PE\left(n\right) \end{array}$$

where $S_{n}$ is the n-th word in a sentence, $w_{1}$ and $b_{1}$ are the weight matrix and bias vector of the first fully connected layer. $w_{2}$ and $b_{2}$ are corresponding parameters of the second layer. The feature vector $\tilde{S}$ equals the product of weight matrix *w* and input vector *S* plus bias *b*. Finally, the positional encoding module (PE) [25] is introduced to retain the order and position information of the sequence.

**Figure 2:**
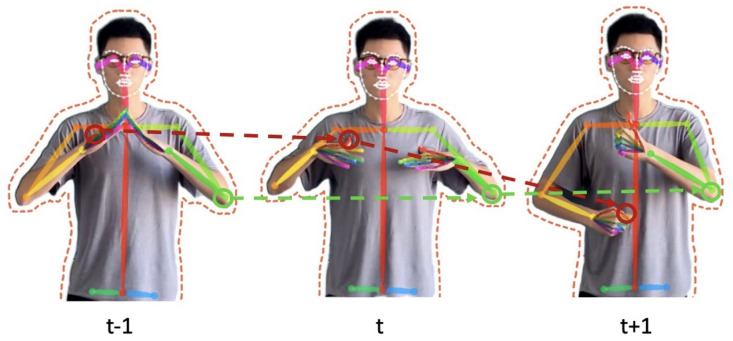
Exploiting Dynamic Information. For example, the red line indicates the motion information of the thumb joint at t−1, *t* and t+1, and the green line indicates the corresponding motion of the elbow joint. The dynamics of joints are defined by the position and velocity information, which implies the spatial displacement of joints with the frame index.

Unlike word embedding, to make a full use of the semantic information of the target sign poses sequence *T*, the RS embedding module extracts the dynamic information with the frame index. Figure 2 shows the details about exploiting dynamic information for adjacent frames. We mark the displacement between adjacent frames as velocity information. Velocity could be seen as both spatial and temporal information of joints. Rich semantics embedding implies the prior information that motion speed of human joints is important. The target sequence is defined as Equation (2). Equation (3) represents position information, and Equation (4) represents velocity information

(2) $$\begin{array}{c} T=(p_{u}^{t},v_{u}^{t})|u=1,2,\ldots ,50\;and\;t=1,2,\ldots ,M \end{array}$$

where

(3) $$\begin{array}{c} p_{u}^{t}=\left(X_{u}^{t},Y_{u}^{t},Z_{u}^{t}\right) \end{array}$$

(4) $$\begin{array}{c} v_{u}^{t}=p_{u}^{t}-p_{u}^{t-1} \end{array}$$

$p_{u}^{t}$ expresses the position information of joint *u* at frame *t* and $v_{u}^{t}$ denotes the velocity information between successive frames. Similar to the word embedding module, we embed the position $p_{u}^{t}$ and velocity $v_{u}^{t}$ in the same way as Equations (5) and (6):

(5) $$\begin{array}{c} \tilde{v_{u}^{t}}=\;relu\left(w_{2}^{v}\left(relu\left(w_{1}^{v}v_{u}^{t}+b_{1}^{v}\right)\right)+b_{2}^{v}\right)+PE\left(t\right) \end{array}$$

(6) $$\begin{array}{c} \tilde{p_{u}^{t}}=\;relu\left(w_{2}^{p}\left(relu\left(w_{1}^{p}p_{u}^{t}+b_{1}^{p}\right)\right)+b_{2}^{p}\right)+PE\left(t\right) \end{array}$$

We map the position and velocity information to the same vector space through neural network. All joints information are fused together at each time. Equation (7) represents the sign poses sequence after embedding.

(7) $$\begin{array}{c} \tilde{T}=(\tilde{p_{u}^{t}},\tilde{v_{u}^{t}})|u=1,2,\ldots ,50\;\mathrm{and}\;t=1,2,\ldots ,M \end{array}$$

### 3.2. Relaxed Mask

Masked mechanism [25] in multi-head self attention was developed in response to prevent the leakage of future information when decoding target sequences. Instead of using the restricted self attention, we create a relaxed masked self attention and set future groups to −∞. Divided level *d* specifies the group size when splitting the target sequence. The total *M* frames can be divided into $\left(\frac{M-1}{d}+1\right)$ groups, and each group contains *d* consecutive frames. When predicting the frames in $Group_{k}$, the relaxed masked mechanism enables the decoder to access to all frames in $Group_{1}$, $Group_{2}$, …, $Group_{k}$. Since our model generates a group of sign poses in parallel, there is no need to mask the frames inside the current group. Given the target frame number *M* and divided level *d*, relaxed mask $m\in R^{M\times M}$ is defined as Equation (8).

(8) $$\begin{array}{c} m\left[i\right]\left[j\right]\left\{\begin{array}{cc} 0 & if\;j<(\frac{i-1}{d}+1)\times d\\ -\infty & otherwise \end{array}\right. \end{array}$$

As a result, the relaxed mask attention with a residual connection is defined in Equation (9).

(9) $$\begin{array}{c} X_{l}=softmax\left(\frac{QK^{T}}{\sqrt{d_{k}}}+m\right)V+X_{l-1} \end{array}$$

where the input consists of query *Q*, key *K*, value *V*, keys of dimension $d_{k}$ and relaxed mask *m*. When calculating the dot product, dividing by $\sqrt{d_{k}}$ is to keep the variance equal to 1. We mask future groups into −∞ by adding *m* to the dot product of *Q* and *K*. The softmax operation normalizes the results so the scores of future groups are changed into 0. Hence the relaxed mask attention mechanism won’t peek on the future groups which means only using the previous positions and the current group to predict.

**Figure 3:**
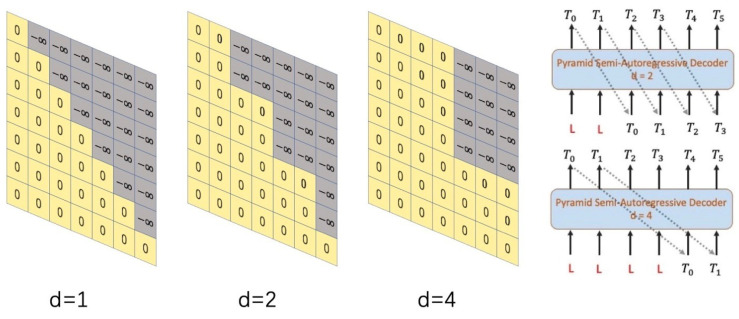
Relaxed mask. When d=1, it is actually equivalent to the restricted self-attention. d=2 means the divided level is 2 and two frames in a group. d=4 means the divided level is 4 and four frames in a group. The vertical axis represents the position of the target vocabulary, and the horizontal axis represents the viewable position.

As shown in Figure 3, the gray part of the matrix is −∞, which represents the masked information, and the yellow part represents the information that is not masked. Matrices with different divided levels are applied to each sequence to achieve the effect of our pyramid semi-autoregressive mechanism.

### 3.3. Pyramid Semi-Autoregressive Transformer

In this work, we propose a Pyramid Semi-Autoregressive transformer for sign language production which uses transformer with a novel decoding manner as the backbone network.

**Text Feature Encoder.** Similar to the autoregressive transformer, the text feature encoder learns semantic features from spoken language sentences. The encoder is composed of *N* blocks with the identical structure but diverse parameters. Each block is composed of two sub-layers, named multi-head attention mechanism (MHA) and position-wise feed-forward network (FFN). Each sub-layer is added with residual connection and layer normalization (LN). The output of sub-layer is shown in Equation (10).

(10) $$\begin{array}{c} SubLayer_{output}=LN\left(\tilde{S}+\left(SubLayer\left(\tilde{S}\right)\right)\right) \end{array}$$

MHA allows the model to jointly process information from different representation subspaces at different locations. It projects query *Q*, key *K* and value *V* through *h* different linear transformations, and finally concatenate *h* different attention results into a single matrix $W^{O}$. MHA can be formulated as Equations (11) and (12).

(11) $$\begin{array}{c} MHA\left(Q,K,V\right)=Concat\left(head_{1},\ldots ,head_{h}\right)W^{O} \end{array}$$

where

(12) $$\begin{array}{c} head_{i}=Attention\left(QW_{i}^{Q},KW_{i}^{K},VW_{i}^{V}\right) \end{array}$$

Assumed *x* is the output after a multi-head attention sub layer, the FFN is applied to each position separately and identically. The formula is shown as Equation (13).

(13) $$\begin{array}{c} FFN\left(x\right)=max\left(0,w_{1}^{FFN}x+b_{1}^{FFN}\right)w_{2}^{FFN}+b_{2}^{FFN} \end{array}$$

**Sign Length Predictor.** Suppose the length of target sign language video is *L* and divided level is *d*, our PSAT mechanism divides target sequences into $\frac{L}{d}$ groups in different decoder blocks. It keeps the left-to-right autoregressive property at group-level but generates *d* frames parallelly inside each group. Therefore, the length of target sign poses is an important latent variable, which strongly affects the ultimate production quality. Inspired by Wang et al. [29], our sign length predictor uses a single-layer neural network with softmax classifier to sum the hidden vectors from the encoder. The maximum length *L* in our experiments equals to 300. There is another loss function to predict the target length with the ground truth length which means not tuning the encoder’s parameters about SLP. Note that we only use the sign length predictor to assist inference during the testing phase. When training the SLP model, the lengths of ground truth sign poses are actually used.

**Pyramid Semi-Autoregressive Decoder.** We introduce a novel PSAT transformer with a group of coarse-to-fine divided levels *d*. It can produce the whole target sequences in a globally serialized and locally parallel manner. When generating the frame *t*, the decoder takes both the sign pose in frame t−d and the products of encoder as input. Note that, we input the length *L* of sign poses sequence at the first *d* frames.

The first layer of decoder is relaxed masked attention. We divide the target sign poses sequence $\tilde{T}$ into $\frac{M-1}{d}+1$ groups. Each group has *d* frames and its conditional probability can be formulated as Equations (14) and (15).

(14) $$\begin{array}{c} P\left(\tilde{T}|\tilde{S}\right)=\prod_{t=1}^{\left[\frac{M-1}{d}\right]+1}P(G_{t}|G_{<t},\tilde{S}) \end{array}$$

where

(15) $$\begin{array}{c} G_{1},G_{2},\ldots ,G_{\frac{M-1}{d}+1}={\tilde{T}}_{1}\ldots {\tilde{T}}_{d},{\tilde{T}}_{d+1}\ldots {\tilde{T}}_{2d},\ldots ,{\tilde{T}}_{\frac{M-1}{d}+1}\ldots {\tilde{T}}_{M} \end{array}$$

$G_{<t}$ represents the groups before *t*-th group. *M* represents the frames in the target sequence after embedding $\tilde{T}$.

We implement our PSAT decoder with the purpose of getting a faster generation than AT, and furthermore, generating more stable sign poses sequence than NAT. In order to balance the inference speed and generation quality, our PSAT is in a coarse-to-fine divided level on each decoder block: d=8, d=4, d=2 and d=1. It should be noted in Figure 3, the divided level in the top block is d=1. In fact, it is equivalent to AT which aims at remaining the correlation between adjacent frames in the final output. The top block focuses on long-distance global semantic information while the bottom block especially concerns short-distance information. Thus, it becomes possible for the decoder to keep longer-term dependency and have a more efficient decoding speed in the meanwhile.

Encoder-Decoder attention layer is used to focus on the appropriate alignment between spoken language sentence and sign pose sequence. Therefore, the structure is similar with the encoder, except creating the query matrix from the output of the previous layer (Feed Forward) and the Key and Value matrices come from the output of the encoder actually. The output of decoder is a vector of floats and we use the final linear layer to turn that into the predicted sequence T. Mean square error loss MSE(T,T) is utilized to fit out model minimizing the error between predicted T and the ground truth *T*.

## 4. Experiments

### 4.1. Datasets and Evaluation Criteria

The experiments begin with both German and Chinese public sign language datasets to demonstrate the effectiveness of our model in SLP. RWTH-PHOENIX-Weather-2014T [30] records the daily news and weather forecast airings of the German public TV-station PHOENIX featuring sign language interpretation. It contains 8257 video samples, and a total of 2887 words are combined into 5356 continuous sentences related to weather forecast. Chinese sign language (CSL) dataset [31] is adopted to validate the generalization ability of our PSAT-RS model for sign languages. The CSL corpus contains 100 sentences and 5000 continuous sign language videos in total. Each sentence contains an average of 4–8 words.

To evaluate the quality of SLP results, we adopt the state-of-the-art transformer based continuous sign language translation (SLT) model [18] as back translation method. According to the baseline method [2], the input of SLT model is changed from sign video frames to sign pose sequences.

The scores are presented by standard metrics including BLEU-1/4 and ROUGE. BLEU measures how much the frames in the generated sign language video appeared in the Ground Truth. ROUGE measures how much the frames in the Ground Truth appeared in our generated sign language video.

### 4.2. Implementation Details

The proposed models are built by PyTorch deep learning framework and a NVIDIA geforce RTX 3060 GPU is used for model training and inference.

**Data preprocessing.** Firstly, sign language videos were processed using Openpose [32] to extract the 2D skeletons. In order to remove redundant information and reduce the amount of calculation, 8 joints of the upper body and 42 joints of both hands were intercepted, with a total of 50 joints. Secondly, 2D to 3D inverse kinematics is used to lift the 2D information to 3D. By observing imbalance data distribution of the 3D sequence, we discarded the abnormal joints and processed the missing joints through weighted linear interpolation.

**Model training.** During the training phase, both our model and compared methods almost follow the same hyper-parameters setting: Embedding=512, Hidden=512, Feedforward=2048, Heads=8, layers=4, Batchsize=32, dropout=0.1. We use Adam [33] as the optimizer and the total number of iterations is 40 k. We use the cosine decay with warmup learning rate [34] lr in Equation (16):

(16) $$\begin{array}{c} lr\left({step}_{global}\right)={lr}_{min}+\frac{1}{2}\times \left({lr}_{max}-{lr}_{min}\right)\times (1+\cos\left(\pi \frac{{step}_{global}-{step}_{warmup}}{{step}_{total}-{step}_{warmup}}\right) \end{array}$$

where we set the maximum initial learning rate $lr_{max}$ as $1\times 10^{-3}$ and the minimum learning rate $lr_{min}$ as $1\times 10^{-4}$. The gradual warmup strategy is employed in the first 100 steps which symbolized as $step_{warmup}$. The current executed step is $step_{global}$ and the total number is $step_{total}$.

**Pre-trained initialization.** In order to accelerate the convergence speed of gradient descent in PSAT and obtain a model with low generalization error, we initialize a part of parameters in the PSAT by transferring the knowledge learned from a pre-trained transformer [25], including all parameters in the text feature encoder, semantics richness embedding layer, and some parameters of the pyramid semi-autoregressive decoder. For other parameters, we conduct random initialization. By this way, the problem of gradient disappearance or explosion caused by improper initialization is avoided. We find this method slightly improves the SLP quality.

### 4.3. Ablation Studies

In this section, we will experimentally analyze PSAT-RS in detail from the following aspects.

**Figure 4:**
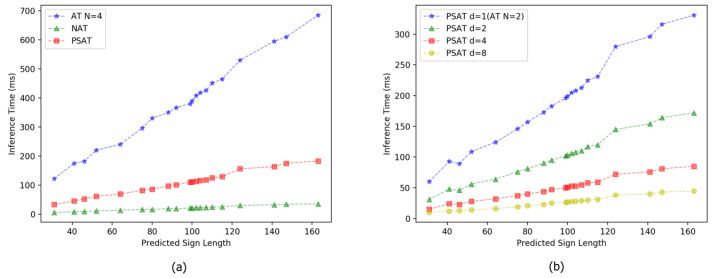
The relationship between inference latency and the predicted sign length in different model configurations. 20 sign poses sequence of length within [31, 163] are randomly selected. (**a**) shows the experimental results of AT, NAT and PSAT. (**b**) shows the experimental results under a single configuration d=1,2,4,8 in PSAT. In order to simplify the calculation, two-layer decoder is selected.

**The optimal pyramid structure of divided levels.** We first vary the number of divided level *d* and then provide a direct comparison to a lighter transformer with two blocks (N=2). d={2,2} means all decoder blocks using relaxed masked attention with d=2. Table 1 summaries speed and precision of PSAT results in different configurations. According to the results, as *d* increases, the BLEU score of the predicted sign sequence gradually decreases. When d={2,2}, the PSAT decodes 1.90× faster than the Transformer and only drops 0.09 BLEU-1 score. With d={8,8}, the PSAT can achieve 7.32× speedup while BLEU-1 score drops by 4.84. Figure 4b presents a direct comparison of coarse-to-fine divided levels. We can find that divided level and inference time are in inversely-proportional relationship. Along with the gradual increase of *d*, the latency becomes gradually shorter.

Moreover, we present the ablation experiments results of the proposed PSAT for SLP on the RWTH-PHOENIX-Weather-2014T dataset. As shown in Table 1, we can find that a group of coarse-to-fine divided levels brings further improvement than a single configuration. d={1,1,1,1} represents the case when divided level of each block is 1, which is actually equivalent to autoregressive transformer. d={8,4,2,1} means that the bottom block uses relaxed masked attention with d=8, the second block with d=4, the third block with d=2, until the top block is in the restricted self-attention which runs autoregressively during inference. d={2,2,2,2} and d={8,6,4,1} are neck-and-neck in BLEU-1 score, however the speedup of the latter is 3.60× which far exceeds the speedup of the former. We also conduct an experiment for d={8,6,4,2}, the speedup is 4.75× but the BLEU-1 score sacrifices too much, which is 2.48 BLEU-1 score less than configuration d={8,4,2,1}. Hence, we set d={8,4,2,1} as the optimal pyramid structure of divided levels.

**The effectiveness of the proposed modules.** To verify the effectiveness of our proposed modules, we gradually embed our two modules into the progressive transformer with gaussian noise (PT & GN) [2]. The results are listed in Table 2 and Table 3.

- The effectiveness of RS embedding worked can be clearly seen in the cases of PT(GN) and PT (GN & RS). When we embed RS into PT(GN), the BLEU-1 and ROUGE scores are improved by 0.15 and 0.38 respectively. It verifies the importance of the combination of position features and motion features.
- In comparison PT (GN) with our PSAT, although the accuracy scores are slightly dropped on DEV SET, it can be observed that the BLEU-1 and ROUGE scores respectively improved by 0.68 and 0.79 on TEST SET, since our method alleviates error accumulation during decoding. Another important thing observed in Table 2 is that our P-SAT speedups 3.6× which efficiently optimizes the inference latency.
- After simultaneously using both our PSAT and RS embedding module, PSAT & RS shows that the BLEU score lifts 0.64 and ROUGE score lifts 0.63, confirming the effectiveness of the proposed algorithm.

### 4.4. Quantitative Evaluation

We compare our PSAT-RS method with several other state-of-the-art models, including PT [2] with gaussian noise and NAT methods [6,7]. Table 2 summaries results of SLP on dataset RWTH-PHOENIX-Weather-2014T. Note that the pre-trained evaluation model in B. Saunders’s work [2] is not publicly available, we train the back translation model based on SLT by ourselves. Although the presented results in their paper are not comparable to ours, we reproduced their results as much as possible and made a relatively fair comparison in the same standard training settings.

**Effects of reducing inference latency.** There are two factors affect the inference efficiency. One is the time complexity of the regression method adopted by the decoder, and the other is the length of the target sequence. In transformer, only one time step is moved each time during inference. We use O(ts) to represent the time complexity of this step. For the probability distribution obtained during inference, we use greedy search to take the possibility of the maximum value from probability distribution of each sign frame, and the time complexity is expressed by O(gs). The time complexity of each model is shown in Table 3, where *L* represents the length of target sign poses sequence, *N* represents the number of layers in decoder, and $\sum d^{*}$ represents the sum of divided levels in pyramid structure. As shown in Figure 4a, the inference latency of AT increases linearly with the increase of prediction sign poses length. The parallelizability of NAT model makes the inference latency independent of the target length, and the speed is accelerated by 18.4×. Our PSAT & RS improves the inference time by 3.6× faster than the AT (PT&RS) without sacrificing the accuracy (even lifts 0.64 BLEU score and 0.79 ROUGE score on TEST SET).

**Effects of improving accuracy.** To explore AT method, we first reproduce the results of PT [2]. In Table 2, after combining our RS embedding, the improved score shows the effectiveness of mining velocity information. At the same time, we set up NAT for comparative experiments to explore the impact of diverse regression methods of the decoder. We refer to the NAT process in Huang et al. [6] and Hwang et al. [7]. Table 2 and Table 3 illustrate that although NAT improves the inference speed, the accuracy is greatly sacrificed. Finally, compared with PT(GN), the PSAT & RS improves 0.91 BLEU score and 1.42 ROUGE score on TEST SET. It proves that our PSAT-RS is both efficient and effective which makes a better trade-off between speed and accuracy.

### 4.5. Qualitative Results

In order to show the performance of the PSAT-RS, we separately compare the generated sign poses sequences by different models on RWTH-PHOENIX-Weather-2014T and CSL datasets. To prevent errors caused by different proportion of human bones, we select 100 different sentences demonstrated by the same signer from CSL dataset. Among them, 70 are used for training, 15 for validation, and 15 for testing. Due to the skeleton information in CSL is redundant, we refer to B. Saunders’s [2] extraction method for RWTH-PHOENIX-Weather-2014T dataset. Each sign language video is processed into the corresponding sign poses sequence of 50 joints. We perform the continuous SLP based on CSL dataset for the first time.

**Figure 5:**
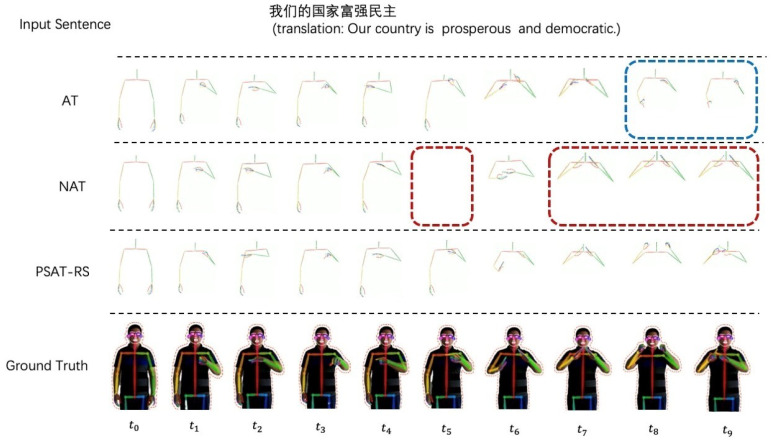
Qualitative results on CSL dataset. We uniformly selected 10 frames for each video.

Figure 5 is the visualization results on CSL dataset. From left to right, we successively sample 10 frames of the sign poses sequence of “our country is prosperous and democratic” for comparison, in which each column represents the frame generated by different models at a certain time. The top row is the result based on the AT model, where $t_{8},t_{9}$ frames have a strong bias towards the ground truth. It is due to the error accumulation caused by excessive dependence between frames. The middle row is the produced frames by NAT, which misses frame $t_{5}$ and generates repeated frames at $t_{7},t_{8},t_{9}$. The above problems due to NAT excessively discard the correlation between sign frames, and outputs them in parallel at each time. Our PSAT-RS result is in the bottom row, which is a more realistic and accurate sign poses sequence.

**Figure 6:**
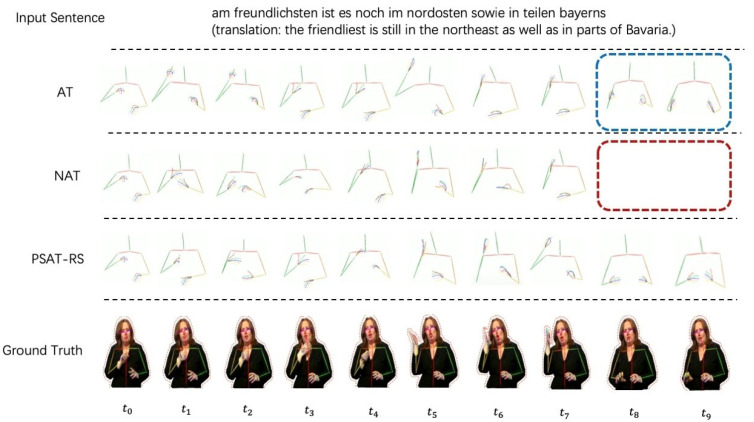
Qualitative results on RWTH-PHOENIX-Weather 2014 T dataset. We uniformly selected 10 frames for each video.

Figure 6 shows the visualization results on RWTH-PHOENIX-Weather-2014T dataset. At $t_{8}$ and $t_{9}$, AT emerges the problems of out-of-true. NAT leads to omission of frames. In comparison, the result in PSAT-RS presents more stable and dynamic sign poses. This demonstrates that our method exploits the nature of semi-autoregressive decoding and thus avoids the shortcomings of AT and NAT.

## 5. Conclusions

In this work, we propose a novel SLP method named PSAT-RS, which aims at accomplishing SLP task in a better trade-off between speed and accuracy. This is the first SLP method which keeps the autoregressive property in global but generates sign poses locally in parallel. A Pyramid Semi-Autoregressive Transformer with coarse-to-fine divided levels is the core of our model. Another significant finding is that velocity information is an important feature and for the first time we use RS embedding module to fuse the sequential information both on time dimension and spatial displacement into the same high-dimensional space. The extensive experiments demonstrate the efficiency and effectiveness of PSAT-RS, which achieve the superior performance on the RWTH-PHOENIX-Weather-2014T and CSL datasets. In the future, we plan to conduct a multimodal learning composed of sign poses, lip moving, and head features.

## Figures and Tables

**Table 1:** Comparison of precision and speed in different configurations on RWTH-PHOENIX-Weather-2014T.

Approach	BLEU-4	BLEU-3	BLEU-2	BLEU-1	ROUGE	Mean Latency (ms)	Speedup
Transformer N = 2	7.09	8.44	12.08	19.20	20.00	192	1×
PSAT d = {2, 2}	6.74	8.13	11.90	19.11	19.76	101	1.90×
PSAT d = {4, 4}	6.17	7.62	11.05	17.21	18.70	50	3.86×
PSAT d = {8, 8}	5.66	7.00	10.22	14.36	16.61	27	7.32×
PSAT d = {1, 1, 1, 1}	11.38	14.59	20.35	32.25	33.09	387	1×
PSAT d = {2, 2, 2, 2}	11.04	14.38	19.92	32.13	32.90	210	1.84×
PSAT d = {8, 6, 4, 2}	10.16	13.37	18.43	29.62	30.51	81	4.75×
PSAT d = {8, 4, 2, 1}	11.02	14.47	20.08	32.10	33.01	108	3.60×

**Table 2:** Comparison of the performance with state-of-the-art models on RWTH-PHOENIX-Weather-2014T.

Approach	DEV SET					TEST SET				
	BLEU-4	BLEU-3	BLEU-2	BLEU-1	ROUGE	BLEU-4	BLEU-3	BLEU-2	BLEU-1	ROUGE
Autoregressive models										
PT (GN) [2]	11.38	14.59	20.35	32.25	33.09	9.45	12.52	17.08	26.59	27.31
PT (GN & RS)	**12.00**	**15.27**	**21.11**	**32.40**	33.47	9.68	12.78	17.28	26.86	28.01
Non-Autoregressive models										
NAT [6,7]	6.90	10.10	13.76	22.76	24.43	4.86	7.11	10.28	18.94	19.97
Our models										
PSAT	11.02	14.47	20.28	32.10	33.01	9.72	12.78	17.35	27.27	28.10
PSAT & RS	11.39	14.85	21.04	32.33	**33.51**	**10.01**	**13.19**	**18.62**	**27.50**	**28.73**

**Table 3:** Comparison of mean latency, speedup, complexity in AT, NAT and PSAT on RWTH-PHOENIX-Weather-2014T.

Approach	Mean Latency (ms)	Speedup	Complexity
AT	387	1×	L(O(ts)+O(gs))
NAT	21	18.4×	O(ts)+O(gs)
PSAT	108	3.60×	$\frac{LN}{\sum d^{*}}\left(O\left(ts\right)+O\left(gs\right)\right)$

## Data Availability

Data are contained within the article and anyone can be used.
